# Video-Oculography-Based Detection of Skew Deviation in Patients Experiencing Dizziness in the Outpatient Setting: A Prospective Diagnostic Accuracy Study

**DOI:** 10.3390/brainsci16080850

**Published:** 2026-08-11

**Authors:** Tzu-Pu Chang, Hong-Hua Lin, Anand K. Bery

**Affiliations:** 1 Department of Neurology/Neuro-Medical Scientific Center, Taichung Tzu Chi Hospital, Buddhist Tzu Chi Medical Foundation, Taichung 427, Taiwan; 2 Department of Neurology, School of Medicine, Tzu Chi University, Hualien 970, Taiwan; 3 Department of Research, Taichung Tzu Chi Hospital, Buddhist Tzu Chi Medical Foundation, Taichung 427, Taiwan; 4 Department of Otolaryngology, Massachusetts Eye and Ear Infirmary, 243 Charles Street, Boston, MA 02114, USA; 5 Department of Otolaryngology, Harvard Medical School, Boston, MA 02114, USA

**Keywords:** skew deviation, dizziness, vertigo, video-oculography, central vestibulopathy, diagnosis

## Abstract

**Highlights:**

**What are the main findings?**

- Skew deviation is uncommon among dizzy patients in outpatient clinics but is highly specific for predicting central lesions.
- The automated video-oculography (VOG) algorithm demonstrates promising performance in detecting skew deviation.

**What are the implications of the main findings?**

- Because of its diagnostic value for identifying central lesions, the test of skew deviation can be incorporated into routine vestibular examinations in outpatient clinics.
- With further refinement, the VOG algorithm may achieve improved accuracy in the automated detection of skew deviation in the future.

**Abstract:**

**Background:** Skew deviation, a vertical ocular misalignment caused by brainstem or cerebellar dysfunction, is strongly associated with central lesions in acute vestibular syndrome but has not been systematically studied in outpatient settings. This clinic-based study evaluated the prevalence of video-oculography (VOG)-guided skew deviation, its diagnostic value for posterior fossa lesions, and the accuracy of an automated VOG algorithm for skew detection. **Methods:** Consecutive dizzy patients attending a neurology clinic over 18 months underwent a VOG-guided alternate cover test. Vertical eye deviation was automatically quantified by the VOG software and validated by expert review of VOG traces and/or videos (expert review constituted the gold standard in this study). We analyzed the prevalence and diagnostic performance of skew deviation for posterior fossa lesions. Receiver operating characteristic analysis and area under the curve (AUC) were used to evaluate the automated algorithm. **Results:** Among 226 dizzy patients, skew deviation was identified in 5.3% (95% CI 3.1–9.1%). For predicting posterior fossa lesions, skew deviation showed a sensitivity of 23.7% (95% CI 13.0–39.2%), specificity of 98.4% (95% CI 95.4–99.5%), positive predictive value of 75.0% (95% CI 46.8–91.1%), and negative predictive value of 86.4% (95% CI 81.2–90.4%). The automated VOG algorithm demonstrated promising performance for detecting skew deviation, with AUCs of 0.88 for the right eye (95% CI 0.77–0.99) and 0.94 for the left eye (95% CI 0.91–0.97). **Conclusions:** Skew deviation is uncommon in outpatient dizzy patients, but its high positive predictive value supports its utility as an adjunctive predictor of posterior fossa lesions. Although the automated VOG algorithm showed promising performance, expert review of traces and/or video remains necessary because false-positive results may occur.

## 1. Introduction

Skew deviation is a vertical ocular misalignment resulting from dysfunction of the vestibular graviceptive pathways [1]. During sustained head tilt in the roll plane, the utricle transmits head-tilt signals through the vestibular nerve, vestibular nuclei, medial longitudinal fasciculus, and oculomotor and trochlear nerves to the extraocular muscles, producing compensatory ocular counter-roll [2,3]. When one side of this graviceptive pathway is disrupted (for example, by a lesion anywhere along the pathway), ocular torsion occurs due to an imbalance in vestibular input. With more severe dysfunction, vertical misalignment of the eyes develops, resulting in skew deviation [4,5].

Although both peripheral and central lesions along the graviceptive pathways can cause skew deviation, peripheral skew deviation is typically small in magnitude and transient, whereas central skew deviation tends to be larger and more persistent [6]. Therefore, in clinical practice, skew deviation is generally considered a central sign (especially when it is seen with alternate cover testing). This concept has been incorporated into the evaluation of vertigo in the emergency setting. The three-step bedside examination HINTS (Head-Impulse, Nystagmus, Test of Skew) is widely used to differentiate posterior fossa stroke from vestibular neuritis [7]. Among its components, skew deviation is modestly sensitive but highly specific [8], making it a valuable adjunctive sign, particularly in cases where the head impulse test may be falsely positive in stroke.

Despite its established role in the emergency setting for assessing acute vestibular syndrome (AVS), skew deviation has not been systematically studied in the outpatient setting. Many vestibular and ocular motor findings that are evident in the acute period (such as spontaneous nystagmus [whether peripheral or central in etiology]) tend to diminish with time and compensation [9]. Therefore, skew deviation may be less reliable as a marker in the subacute and chronic period, but this has not been studied, and the prevalence of skew deviation among dizzy patients in outpatient clinics remains unclear. It is also unknown whether skew deviation can reliably predict brainstem or cerebellar lesions in this population. Furthermore, recent studies have validated video-oculography (VOG)-based skew deviation measurement against bedside measurement with prisms [10], and applied VOG to quantify skew deviation in emergency settings [11]. Given that VOG is routinely used in outpatient clinics for the evaluation of dizziness, it remains uncertain whether skew deviation testing can be feasibly integrated into standard VOG examinations. In addition, it is unclear whether automated algorithms embedded in VOG systems can accurately detect skew deviation.

Accordingly, this study aimed (1) to determine the prevalence of skew deviation among dizzy patients in outpatient clinics (with VOG), (2) to assess the diagnostic accuracy of skew deviation for predicting brainstem or cerebellar lesions in this setting, and (3) to evaluate automated VOG algorithms in detecting skew deviation under routine practice conditions.

## 2. Materials and Methods

### 2.1. Study Population

We prospectively recruited consecutive patients presenting with dizziness from a neurology clinic at a university-affiliated general hospital between 2024 and 2025. As this was a consecutive observational study, the sample size was determined by the number of eligible patients presenting during the study period; therefore, no a priori sample size calculation was performed. All patients underwent structured history taking, bedside neurological and neuro-otological examinations, and VOG, including gaze testing, saccades, smooth pursuit, optokinetic testing, fixation suppression, and video head impulse testing. Patients with focal neurological signs, central vestibular signs, or central ocular motor abnormalities, including skew deviation, underwent brain MRI to exclude central lesions. We similarly obtained MRI in patients with abnormal gait, or those who had spontaneous nystagmus in the face of normal peripheral vestibular function (i.e., had normal video head impulse testing).

Patients who were blind in one or both eyes were excluded. To ensure that all included patients were symptomatic at the time of assessment, we excluded patients with episodic vestibular syndromes (e.g., vestibular migraine, Ménière’s disease) in the interictal phase. We also excluded patients with positional vertigo (e.g., benign paroxysmal positional vertigo) who were asymptomatic during the alternate cover test in the upright position.

This study was conducted in accordance with the Declaration of Helsinki and was approved by the Institutional Review Board of the Research Ethics Committee of Taichung Tzu Chi Hospital (REC113-06). Written informed consent was obtained from all participants.

### 2.2. VOG-Guided Alternate Cover Test

A well-trained examiner (HHL) performed a VOG-guided alternate cover test to detect skew deviation in all included patients. A binocular VOG system (VertiGoggles, ZT-VNG-II; Shanghai ZEHNIT Medical Technology Co., Ltd., Shanghai, China) was used. This system consists of goggles equipped with two infrared video cameras positioned temporally and software that automatically calculates vertical deviation angles. The frame rate was 60 fps.

After donning the goggles and completing calibration, patients were instructed to fixate on a visual target (a red dot, 1 cm in diameter) positioned 120 cm in front of them. The examiner alternately occluded the right and left eyes using an eye occluder, with each occlusion lasting approximately 3 s. The total examination time for each patient was 30 s. Eye movement videos were recorded, and corresponding VOG traces were automatically generated by the VOG software (VertiPACS version 7.1.0, ZEHNIT).

### 2.3. Algorithm for the Automatic Detection of Skew Deviation

The VOG software automatically calculated the vertical deviation angles of the right and left eyes during the alternate cover test to quantify skew deviation. The algorithm first identified the median vertical eye position over the 30-s recording as the reference point (M0). It then determined the median of the vertical eye positions above M0 (M1) and the median of those below M0 (M2). The vertical deviation angle was defined as the difference between these two values (M1 − M2). The vertical deviation angles for the right and left eyes were calculated separately.

### 2.4. Validation for the Presence or Absence of Skew Deviation

**Figure 1:**
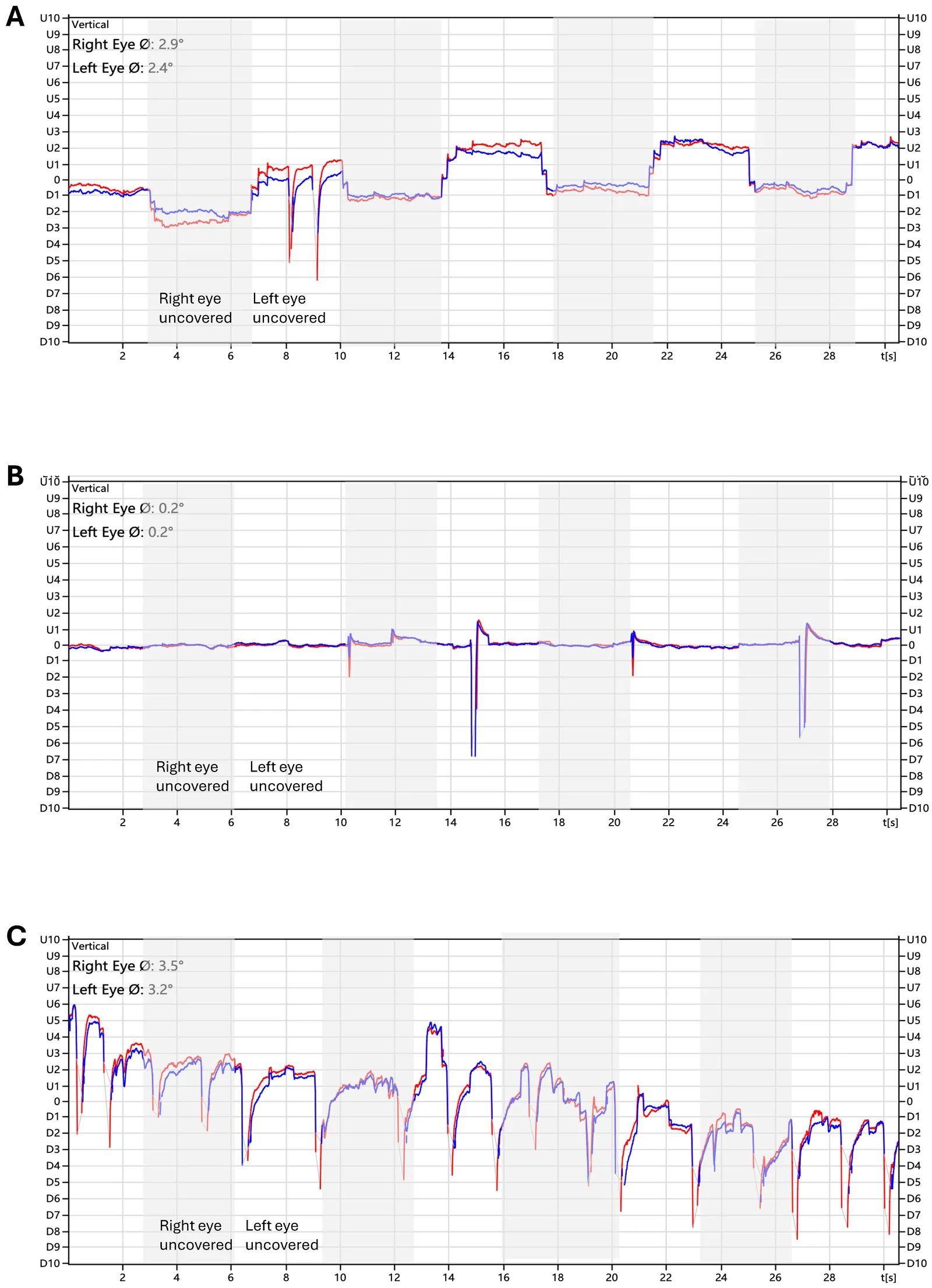
Representative VOG recordings of vertical eye movements during the alternate cover test (red trace represents the right eye position; blue trace represents the left eye position). Gray shading indicates the time during which the right eye is uncovered. In a patient with skew deviation, the vertical VOG trace shows a characteristic repetitive square-wave pattern, with vertical deviation angles of 2.9° in the right eye and 2.4° in the left eye (**A**). In a patient without skew deviation, the VOG trace is flat and does not exhibit a square-wave pattern (**B**). Similarly, another patient without skew deviation also does not exhibit a square-wave pattern; however, blinking artifacts resulted in falsely elevated deviation angles (3.5° in the right eye and 3.2° in the left eye). Clinicians should be aware of this potential source of false-positive results (**C**).

A subspecialty-trained oto-neurologist (TPC), who was blinded to lesion status, graded each VOG trace in this study, confirming presence or absence of skew deviation. This served as the gold standard for classifying skew deviation in the present study, and consisted of manual review of the trace by an expert confirmed a characteristic repetitive square-wave pattern in the VOG traces during alternate cover testing (Figure 1A). In contrast, the absence of such square-wave patterns was considered indicative of no skew deviation (Figure 1B,C). In cases where the VOG findings were equivocal, the corresponding video recordings were comprehensively reviewed to determine the presence or absence of skew deviation. More specifically, to validate the accuracy of the pupil-tracking process of VOG, we reviewed the videos of all patients who exhibited a typical square-wave pattern on VOG during the alternate cover test. In addition, we randomly reviewed the videos of 12 patients who did not exhibit a square-wave pattern on VOG. The video recordings confirmed vertical eye movements during the alternate cover test in all patients with square-wave patterns and showed no vertical eye movements in the 12 patients without square-wave patterns.

### 2.5. Statistical Analysis

Descriptive statistics were used to summarize the demographic data. Parametric data were presented as mean ± standard deviation (SD), whereas nonparametric data were presented as median and interquartile range (IQR). The prevalence of skew deviation among dizzy outpatients was calculated based on the results of the expert review of VOG (i.e., grading of skew vs. no skew).

To assess the performance of the VOG algorithm in automatically detecting skew deviation, the Mann–Whitney U test was used to compare the vertical deviation angles of the right and left eyes during the alternate cover test between patients with and without skew deviation (where expert grading was the gold-standard). Receiver operating characteristic (ROC) analysis and the area under the curve (AUC) were used to evaluate the diagnostic accuracy of vertical deviation angles. The optimal cutoff value was determined, and a higher cutoff of 3.3° was additionally applied to examine changes in sensitivity and specificity.

Finally, to evaluate the performance of skew deviation in predicting posterior fossa lesions, sensitivity, specificity, positive predictive value, and negative predictive value were calculated. All statistical analyses were performed using SPSS version 23 (IBM Corp., Armonk, NY, USA).

## 3. Results

### 3.1. Demographic Data and Prevalence of Skew Deviation in Dizzy Outpatients

**Figure 2:**
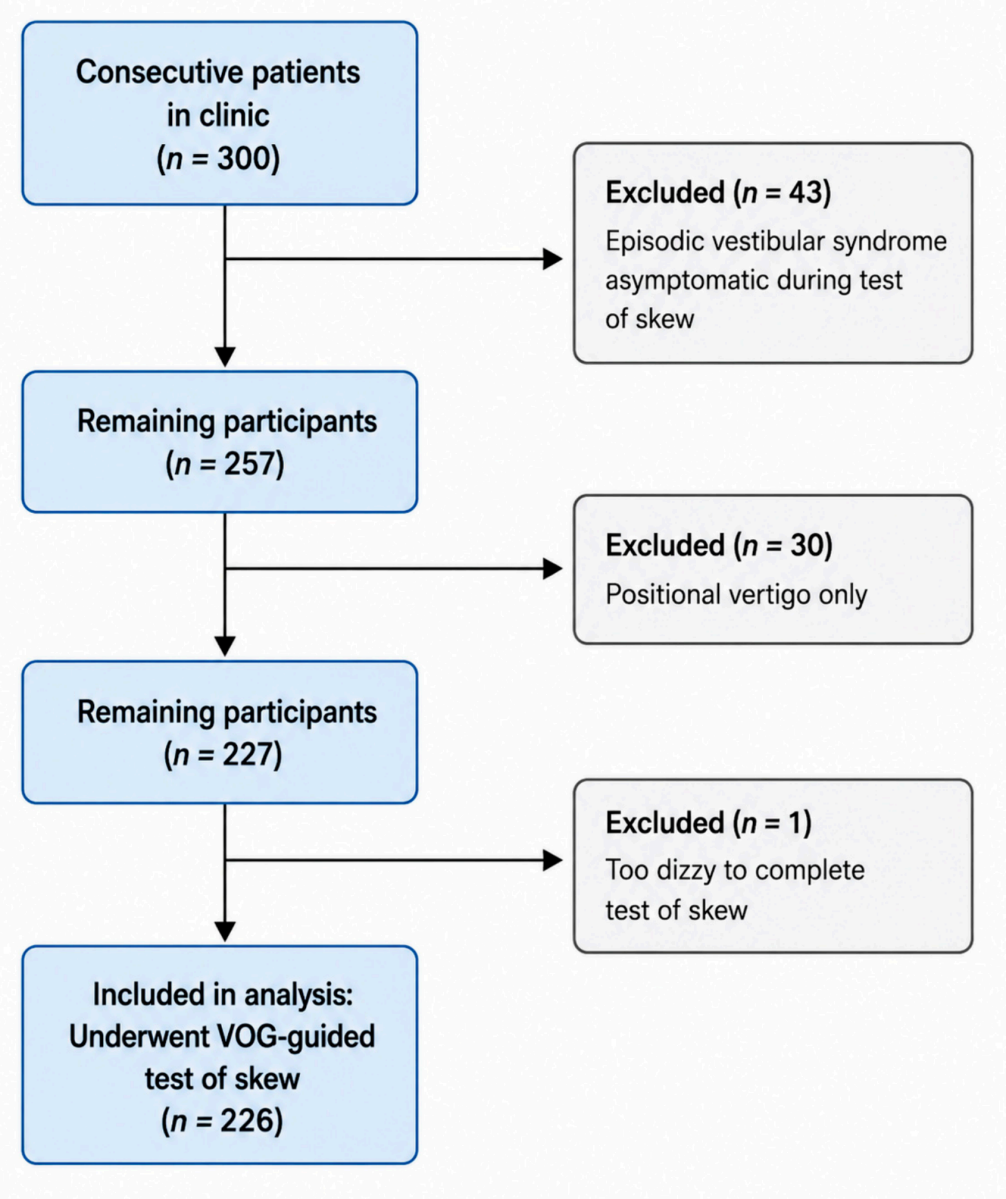
Participant Flow Diagram.

Three hundred consecutive clinic patients were considered for inclusion. Patients with episodic vestibular syndrome who were asymptomatic at time of assessment (*n* = 43) and those with positional vertigo only (*n* = 30) were excluded. One patient was too dizzy to complete testing. This left a total of 226 included patients who underwent VOG-guided test of skew, of whom 137 (60.6%) were female (see Figure 2 for participant flow diagram). No patients were blind in one eye. The mean ± SD age was 64.4 ± 13.1 years (range, 25–94 years). Thirty-eight patients (16.8%) had posterior fossa lesions, including 21 with brainstem lesions, 6 with cerebellar lesions, and 11 with both brainstem and cerebellar involvement. The etiologies comprised vascular causes (*n* = 26), tumors (*n* = 4), degenerative disorders (*n* = 5), and other conditions (*n* = 3; one demyelinating disorder and two Chiari malformations).

Among the remaining 188 patients without posterior fossa lesions, 66 had unilateral vestibulopathy, 18 had bilateral vestibulopathy, 70 had persistent postural-perceptual dizziness (PPPD), and 27 had other diagnoses, including multisensory dizziness (*n* = 13), post-concussion dizziness (*n* = 4), light/heavy cupula syndrome (*n* = 2), carotid stenosis (*n* = 2), parkinsonism (*n* = 3), and cerebral small vessel disease (*n* = 3). Seven patients had an uncertain diagnosis.

During validation, four patients with equivocal VOG findings were classified as not having skew deviation based on video review. Ultimately, 12 patients were identified as having skew deviation, corresponding to a prevalence of 5.3% (95% confidence interval [CI] 3.1–9.1%) among outpatients presenting with dizziness.

### 3.2. Accuracy of Quantitative Analysis for Automatically Detecting Skew Deviation

**Figure 3:**
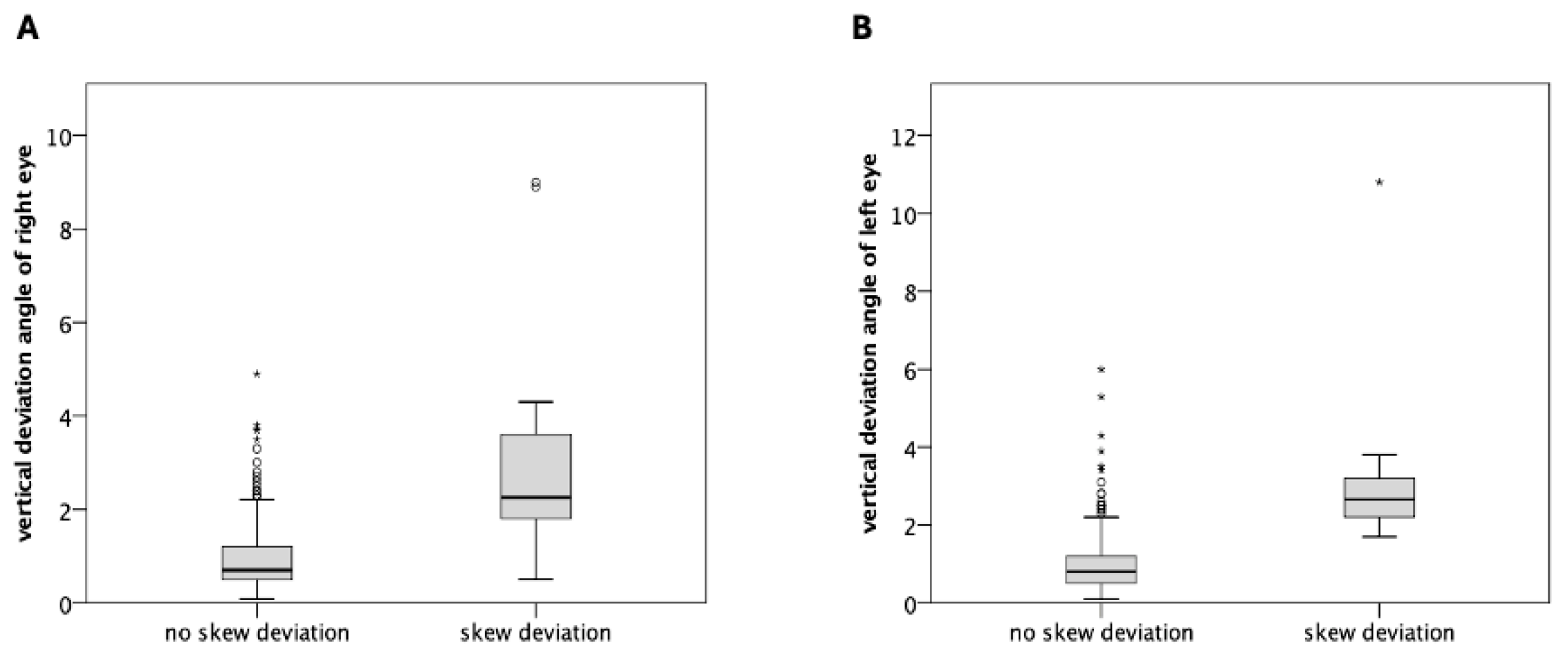
Vertical deviation angles of the right (**A**) and left (**B**) eyes, respectively, during the alternate cover test in patients with and without skew deviation. Data are presented as median (central line), IQR (box), range (whiskers), and outliers.

Among patients with skew deviation (as determined by the validation process explained above), the median (IQR) vertical deviation angle during the alternate cover test was 2.3° (2.2°) for the right eye and 2.7° (1.1°) for the left eye. In contrast, patients without skew deviation had median deviation angles of 0.7° (0.7°) for the right eye and 0.8° (0.7°) for the left eye (Figure 3). For both eyes, deviation angles were significantly greater in patients with skew deviation (*p* < 0.001, Mann–Whitney test).

**Figure 4:**
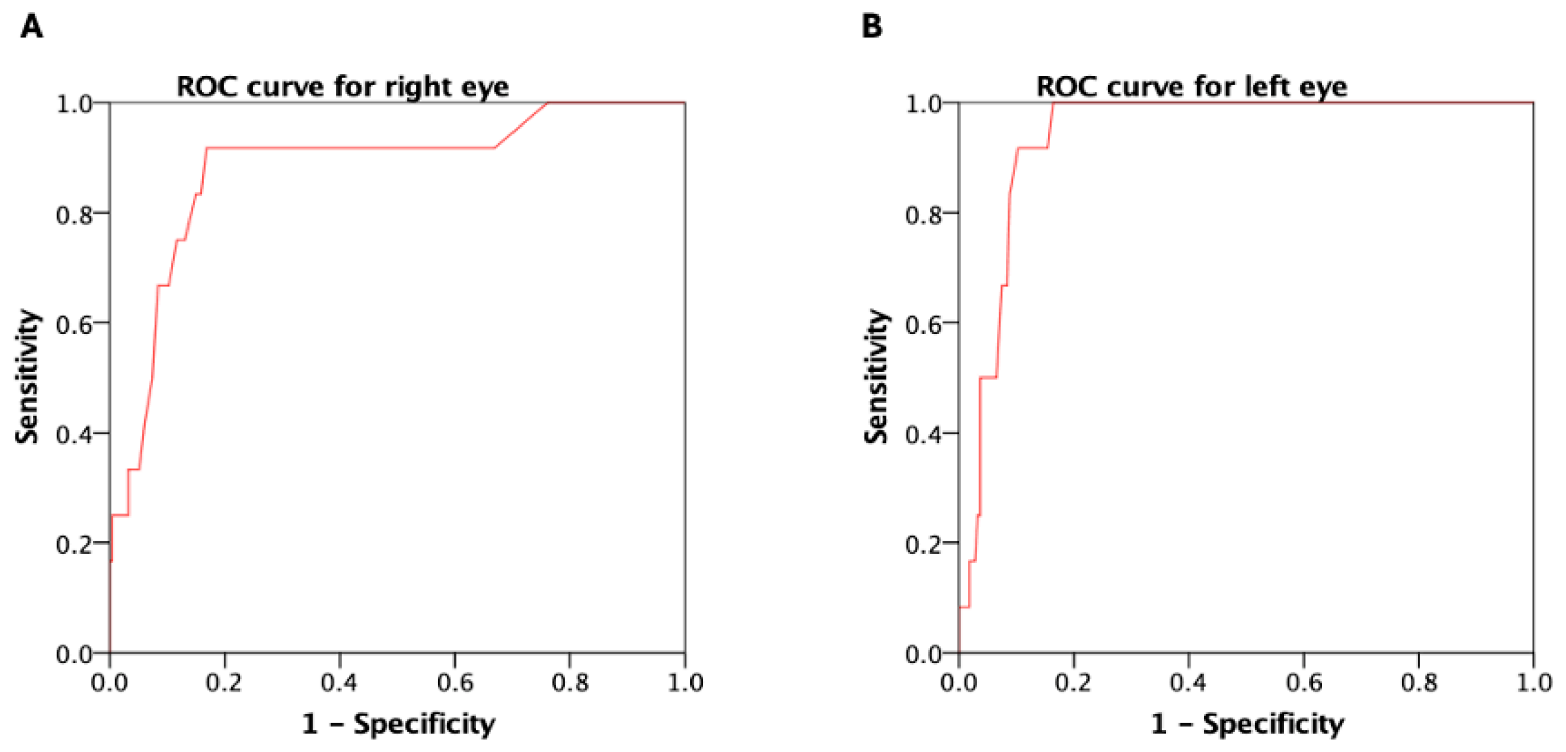
ROC curves for the automatic detection of skew deviation based on the vertical deviation angles of the right (**A**) and left (**B**) eyes, respectively, during the alternate cover test. The AUC was 0.88 for the right eye and 0.94 for the left eye.

ROC analysis showed that using the right-eye vertical deviation angle to detect skew deviation yielded an AUC of 0.88. The optimal cutoff was 1.45° (approximately 2.5 prism diopters), with an apparent (in-sample) sensitivity of 91.7% (95% CI 64.6–98.5) and specificity of 83.2% (95% CI 77.6–87.6%). Unsurprisingly, using a higher cutoff of 3.3° (close to 6 prism diopters) resulted in a sensitivity of 25.0% (95% CI 8.9–53.2%) and specificity of 97.2% (95% CI 94.0–98.7%) (Figure 4A).

Similarly, using the left-eye deviation angle yielded an AUC of 0.94. The optimal cutoff was 1.65°, with a sensitivity of 100% (95% CI 75.8–100.0%) and specificity of 83.6% (95% CI 78.1–88.0%). At a higher cutoff of 3.3°, the sensitivity was 25.0% (95% CI 8.9–53.2%) and specificity was 96.7% (95% CI 93.4–98.4%) (Figure 4B).

### 3.3. Accuracy of Skew Deviation for Predicting Posterior Fossa Lesions

Among the 12 patients with skew deviation, 9 had posterior fossa lesions, yielding a positive predictive value of 75.0% (95% CI 46.8–91.1%). Among the remaining cases, 2 (16.7%, 95% CI 4.7–44.8%) were diagnosed with vestibular neuritis (one had testing in the subacute phase, and demonstrated posterior semicircular canal involvement; while the other had testing 3 months from onset, and demonstrated posterior semicircular canal sparing). One patient (8.3%, 95% CI 1.5–35.4%) had an uncertain diagnosis. The clinical features of the 12 patients with skew deviation is available in Table 1.

Of the 38 patients with posterior fossa lesions, 9 exhibited skew deviation, corresponding to a sensitivity of 23.7% (95% CI 13.0–39.2%). The diagnoses among these nine patients with skew deviation were cerebrovascular disease (*n* = 6), Chiari malformation (*n* = 1), and cerebellar degeneration (*n* = 2). Among the 188 patients without posterior fossa lesions, only 3 had skew deviation, yielding a specificity of 98.4% (95% CI 95.4–99.5%). The negative predictive value was 86.4% (95% CI 81.2–90.4%) (Table 2).

Interestingly, the vertical deviation angles in the 9 patients with posterior fossa lesions (median: 2.2° in the right eye and 2.4° in the left eye) were not greater than those in the 3 patients without posterior fossa lesions (median: 8.9° in the right eye and 2.9° in the left eye).

## 4. Discussion

Although skew deviation has been recognized as a vestibular sign for decades, our study is the first to report its prevalence among dizzy patients in outpatient clinics. We found that skew deviation is relatively uncommon in this setting, with a prevalence of 5.3%. This low prevalence raises the question of whether routine testing for skew deviation is warranted in dizziness clinics. Despite its low frequency, its high specificity and positive predictive value indicate that the presence of skew deviation strongly suggests a central lesion. Moreover, the alternate cover test requires only approximately 30 s to perform, making it a practical addition to the routine evaluation of dizzy patients. Our study supports the idea that the alternate cover test is worth incorporating into routine bedside vestibular examinations and/or as part of standard VOG assessments, though it is limited by low sensitivity and is best interpreted in the context of the full ocular motor and vestibular exam [12]. We therefore recommend its use (whether by conventional methods like alternate-cover, or by VOG) as a *complementary* tool, until independent validation in future studies. In contrast, more detailed but time-consuming examinations, such as the single Maddox rod test [13], may be reserved for selected cases when time efficiency is a consideration.

In the outpatient setting, the sensitivity of skew deviation for detecting brainstem or cerebellar lesions (23.7%) appears lower than that reported in patients with AVS in the emergency department [11]. We acknowledge that this sensitivity value (23.7%) may be an overestimate, given posterior fossa lesions in non-imaged patients could have been missed in this study. However, even in AVS, the sensitivity of skew deviation for detecting central vestibular disorders is only approximately 30% [8,11]. Accordingly, within the HINTS battery, skew deviation is regarded as an adjunctive rather than a primary diagnostic sign. Following the acute stage, ocular tilt reaction tends to resolve over time due to vestibular compensation [14]. Given that most stroke patients presenting to outpatient clinics are beyond the acute phase, it is not surprising that skew deviation is less frequently observed in this population. Interestingly, among the nine patients with central lesions and skew deviation in our study, six had cerebrovascular disease, while three had chronic progressive disorders, including one with Chiari malformation and two with cerebellar degeneration. This finding suggests that skew deviation may also have diagnostic value in progressive or degenerative conditions. While skew deviation is classically associated with lesions of the graviceptive pathway, the cerebellum adjusts/modulates graviceptive processing, and cerebellar lesions are associated with ocular tilt reaction [15] and skew deviation [16]. For this reason, lesions of both the cerebellum and brainstem were considered relevant in this study.

On the other hand, the positive predictive value of skew deviation for detecting brainstem or cerebellar lesions was 75%, indicating that central pathology should be strongly suspected when skew deviation is present. However, skew deviation is not exclusively indicative of central lesions. Among the three patients with skew deviation and negative MRI findings, two were diagnosed with unilateral vestibulopathy. Although not typical, peripheral vestibulopathy has been reported to be associated with skew deviation [6,17]. In a recent AVS study, up to 24% of patients with acute unilateral vestibulopathy exhibited skew deviation, albeit with smaller amplitudes [11]. In contrast, in our study, only 2 of 66 patients (3.0%) with unilateral vestibulopathy demonstrated skew deviation. This discrepancy suggests that peripheral-type skew deviation is rapidly compensated after the acute phase and is therefore less frequently observed in outpatient settings. The remaining patient with an uncertain diagnosis showed a large vertical deviation angle of up to 8.9°. It should be noted that the alternate cover test is not specific for skew deviation and may be positive in other forms of hypertropia, such as congenital strabismus or trochlear nerve palsy (unilateral or bilateral). This issue is particularly relevant when screening large numbers of patients in outpatient clinics. Furthermore, although a recent study of patients with AVS in the emergency department reported that peripheral vestibulopathy was associated with smaller magnitude skew deviations than central vestibulopathy [8], in our study, deviation angles among patients *without* posterior fossa lesions were not smaller than those *with* posterior fossa lesions (considering patients with skew deviation). However, our sample size was too small to draw definitive conclusions.

Our study demonstrates that automated VOG algorithms measuring vertical eye deviation during the alternate cover test has promising performance in detecting skew deviation, with AUC values of 0.88 for the right eye (95% CI 0.77–0.99) and 0.94 for the left eye (95% CI 0.91–0.97). However, because the algorithm cannot fully exclude artifacts related to vertical eye movements, false-positive results may still occur (see Figure 1B). Therefore, reliance on automated VOG analysis alone is insufficient for diagnosing skew deviation. Careful inspection of VOG traces remains necessary, including identification of characteristic square-wave patterns and, when needed, review of the corresponding video recordings. In the future, further refinement of automated algorithms, potentially incorporating artificial intelligence, may enable fully automated detection of skew deviation. In that case, detection of skew in the outpatient setting will be even simpler and more time-efficient, while depending less on examiner skill. The recent study in AVS suggested that a vertical deviation angle of 3.3° is an optimal cutoff for identifying central lesion-related skew deviation [11]. We did not observe similar findings, likely due to the limited number of cases with skew deviation in our cohort, and given our study took place in the outpatient setting. Larger studies are needed to validate these findings.

Several limitations should be acknowledged. First, although patients with concerning central findings on their neurological, vestibular, or ocular motor examinations—including those with skew deviation—underwent brain MRI, not all patients received imaging. Therefore, a small proportion of patients without apparent signs may still have had undetected brainstem or cerebellar lesions, leading to an overestimation of sensitivity. (conversely, measures of positive predictive value and specificity are comparatively protected, since all patients with skew deviation were imaged/verified). Second, as noted above, the alternate cover test may detect hypertropia unrelated to skew deviation. This means our reported prevalence of 5.3% is an upper bound on the true prevalence of skew deviation in the study population. Third, despite enrolling 226 patients, only 12 exhibited skew deviation, limiting the statistical power for subgroup analyses. Fourth, the use of a single examiner and the lack of interrater reliability assessment may limit the reproducibility and generalizability of the study. Fifth, when validating the accuracy of the automated algorithm, we did not use independent measures as the reference standard, and the optimal cutoff values were derived and evaluated using the same dataset. External validation in an independent cohort is required before these cutoff values can be generalized. Lastly, our study population was derived from a tertiary neurology dizziness clinic with a relatively high prevalence of posterior fossa lesions and an older patient population. Therefore, the study results should not be directly extrapolated to primary care or other settings with lower disease prevalence.

## 5. Conclusions

In conclusion, although skew deviation is uncommon among dizzy patients in outpatient clinics, its presence was 98.4% specific for a central lesion in our study (with three false positives), and it therefore remains a valuable adjunctive sign for identifying brainstem or cerebellar lesions. Although limited by low sensitivity when used in isolation, the alternate cover test is simple, rapid, and practical, making it a useful addition to a comprehensive vestibular examination and VOG assessment. While automated VOG algorithms demonstrate promising performance, artifacts remain a limitation; therefore, manual inspection of VOG traces is still required in the current generation of systems. Future work leveraging artificial intelligence-based detection may bring reliable automated detection, one day making skew deviation detection faster and more efficient.

## Figures and Tables

**Table 1:** Clinical features of the patients with skew deviation.

Case No.	Age	Gender	Clinical Features	Diagnosis
1	53	M	vertigo, unsteadiness, right-beating and torsional nystagmus, ocular dysmetria	left dorsolateral medullary infarction
2	68	M	gait ataxia, scanning speech, limb dysmetria, hypometric saccades, saccadic pursuit	multiple system atrophy, cerebellar type
3	61	M	vertigo, vomiting, unsteadiness	bilateral pontine infarction (R > L)
4	47	F	cough-induced dizziness, unsteadiness, gaze-evoked nystagmus	Chiari malformation
5	54	M	vertigo, unsteadiness, right-beating and downbeat nystagmus	left dorsal medullary infarction
6	92	F	vertigo, unsteadiness, abnormal head impulse test	right vestibular neuritis
7	54	F	vertigo, unsteadiness, abnormal head impulse test	right vestibular neuritis
8	78	F	vertigo, unsteadiness, diplopia, internuclear ophthalmoplegia	left pontine infarction
9	61	M	dizziness, unsteadiness, diplopia, oculopalatal tremor	pontine hemorrhage
10	70	M	dizziness, unsteadiness, side-pocket nystagmus	cerebellar degeneration
11	54	F	vertigo, headache, left-beating nystagmus	uncertain
12	62	F	headache, dizziness, unsteadiness	right midbrain cavernous hemangioma with bleeding

M, male; F, female.

**Table 2:** Diagnostic accuracy for skew deviation to predict posterior fossa lesions.

		Posterior fossa lesions		
		+	−	
Skew deviation	+	9	3	Positive predictive value 75.0% (95% CI 46.8–91.1%)
	−	29	185	Negative predictive value 86.4% (95% CI 81.2–90.4%)
		Sensitivity 23.7% (95% CI 13.0–39.2%)	Specificity 98.4% (95% CI 95.4–99.5%)	

## Data Availability

Data are available from the corresponding author on reasonable request.

## Abbreviations

The following abbreviations are used in this manuscript:

HINTS	Head Impulse, Nystagmus, and Test of Skew
AVS	Acute vestibular syndrome
VOG	Video-oculography
SD	Standard deviation
IQR	Interquartile range
ROC	Receiver operating characteristic
AUC	Area under the curve
